# The impact of autoimmune diseases on delirium risk in critically ill patients: a propensity score matching multicenter analysis

**DOI:** 10.3389/fmed.2025.1621441

**Published:** 2025-07-16

**Authors:** Shi-Tao Huang, Kai-Hua Yu, Jing-Wen Yuan, Yi-Bo Sun, Zhong-Ya Huang, Li-Ping Liu

**Affiliations:** ^1^The First Clinical Medical College of Lanzhou University, Lanzhou, China; ^2^Department of Emergency Critical Care Medicine, The First Hospital of Lanzhou University, Lanzhou, Gansu, China

**Keywords:** autoimmune diseases, delirium, propensity score matching, risk factors, intensive care management

## Abstract

**Background:**

Delirium, an acute neuropsychiatric syndrome characterized by disturbances in attention, cognition, and consciousness, is a prevalent manifestation of acute brain dysfunction among intensive care unit (ICU) patients. It is considered within the mental health framework as a transient but serious disorder of cognition and behavior. Autoimmune diseases (AID), characterized by systemic inflammation and immune dysregulation, may impact central nervous system function. Currently, their role in delirium pathogenesis among ICU patients remains unclear. This study aimed to evaluate the association between autoimmune diseases and delirium incidence in ICU patients.

**Methods:**

Using the eICU Collaborative Research Database, we identified patients with first ICU admissions and documented assessment of delirium. Patients were categorized into AID and non-AID groups. Propensity score matching (PSM) and inverse probability weighting (IPTW) were applied to balance key baseline covariates, including demographics, comorbidities, clinical interventions, and severity scores. The primary outcome was delirium occurrence. The association between AID and the occurrence of ICU delirium was evaluated using Cox proportional hazards and competing risk models, with sensitivity and subgroup analyses to assess the stability of the results.

**Results:**

Among 8,978 patients (1,007 with AID; 7,971 without), delirium occurred in 29.7% of the cohort. In both crude and matched cohorts, AID was significantly associated with increased delirium risk in univariate and multivariable Cox analyses (*p* < 0.001). Fine and Gray models confirmed a higher delirium incidence in the AID group after accounting for competing risks of in-ICU mortality (*p* < 0.001). The KM curves show no significant difference in-ICU mortality rate between the two groups.

**Conclusion:**

This study found a significant correlation between AID and the incidence of delirium in ICU, emphasizing the need for heightened delirium surveillance and early intervention in AID patients.

## 1 Introduction

Delirium is a common manifestation of acute brain dysfunction in critically ill patients and is defined as a rapidly developing disturbance in attention, along with fluctuating alterations in consciousness and cognition (1–3). The overall incidence of delirium among hospitalized elderly patients is nearly one-quarter (4), with rates ranging from approximately 20 to 50% in ICU patients, and reaching as high as 60–80% in those receiving mechanical ventilation (5–7). Delirium is associated with a variety of adverse clinical outcomes, including prolonged hospital stay, increased duration of mechanical ventilation, and higher in-hospital mortality (5, 7).

Autoimmune diseases (AID) represent a group of disorders in which the immune system fails to properly recognize self-antigens, resulting in immune-mediated damage to healthy tissues. These aberrant immune responses lead to chronic inflammation and multi-organ injury, affecting various organ systems, with an estimated global prevalence of at least 5% (8–10).

In recent years, studies have increasingly suggested a close association between AID—characterized by systemic inflammation and immune dysregulation—and central nervous system (CNS) dysfunction (11–13). This can manifest clinically as cognitive impairment and neuropsychiatric symptoms. For example, postoperative cognitive dysfunction (POCD) is a frequently encountered neurocognitive complication, particularly among elderly individuals and those with pre-existing frailty or neurodegenerative vulnerability (14, 15). Research has found that in some patients, disease-related pathogenic autoantibodies can be detected, which may directly damage the CNS and lead to significant neurological deficits (12, 13, 16). Additionally, alterations in neuronal innervation have also been implicated (17).

Given these shared pathophysiological mechanisms—including systemic inflammation, autoantibody production, and possible disruption of blood–brain barrier integrity—patients with AID may be particularly susceptible to delirium during ICU stays (18). However, data directly studying this association remain scarce.

Therefore, we conducted a retrospective cohort study using the eICU Collaborative Research Database (eICU-CRD) to investigate whether AID are independently associated with an increased risk of ICU delirium. We applied propensity score matching (PSM) to balance key baseline covariates (including demographics, comorbidities, clinical interventions, and severity scores) and assess this relationship. This study aims to fill this knowledge gap by providing real-world evidence from a large, multi-center ICU cohort and to explore whether AID status may serve as a clinically relevant risk factor for delirium.

## 2 Methods

### 2.1 Data source

This retrospective cohort study was conducted using the eICU-CRD (version 2.0), an open-access clinical database that contains high-granularity data from over 200,000 ICU admissions across 335 ICUs in 208 U. S. hospitals from 2014 to 2015 (19). Access to the eICU-CRD requires researchers to complete a credentialing process that includes passing a training course and agreeing to the data use agreement governed by the PhysioNet Credentialed Health Data Use Committee. The database is released under the HIPAA Safe Harbor provision, ensuring compliance with privacy standards. Approval for data access requires completion of the Collaborative Institutional Training Initiative (CITI) program. Due to the retrospective nature of the study, the Institutional Review Board (IRB) of the Massachusetts Institute of Technology waived the need of obtaining informed consent (Record ID: 58312537). The need to obtain ethical approval was also waived by the Institutional Review Board of the Massachusetts Institute of Technology. The data are certified under the Privacert model to minimize the risk of re-identification. All methods were carried out in accordance with relevant guidelines and regulations. This study followed the Strengthening the Reporting of Observational Studies in Epidemiology (STROBE) guidelines (20).

### 2.2 Study population and design

**Figure 1:**
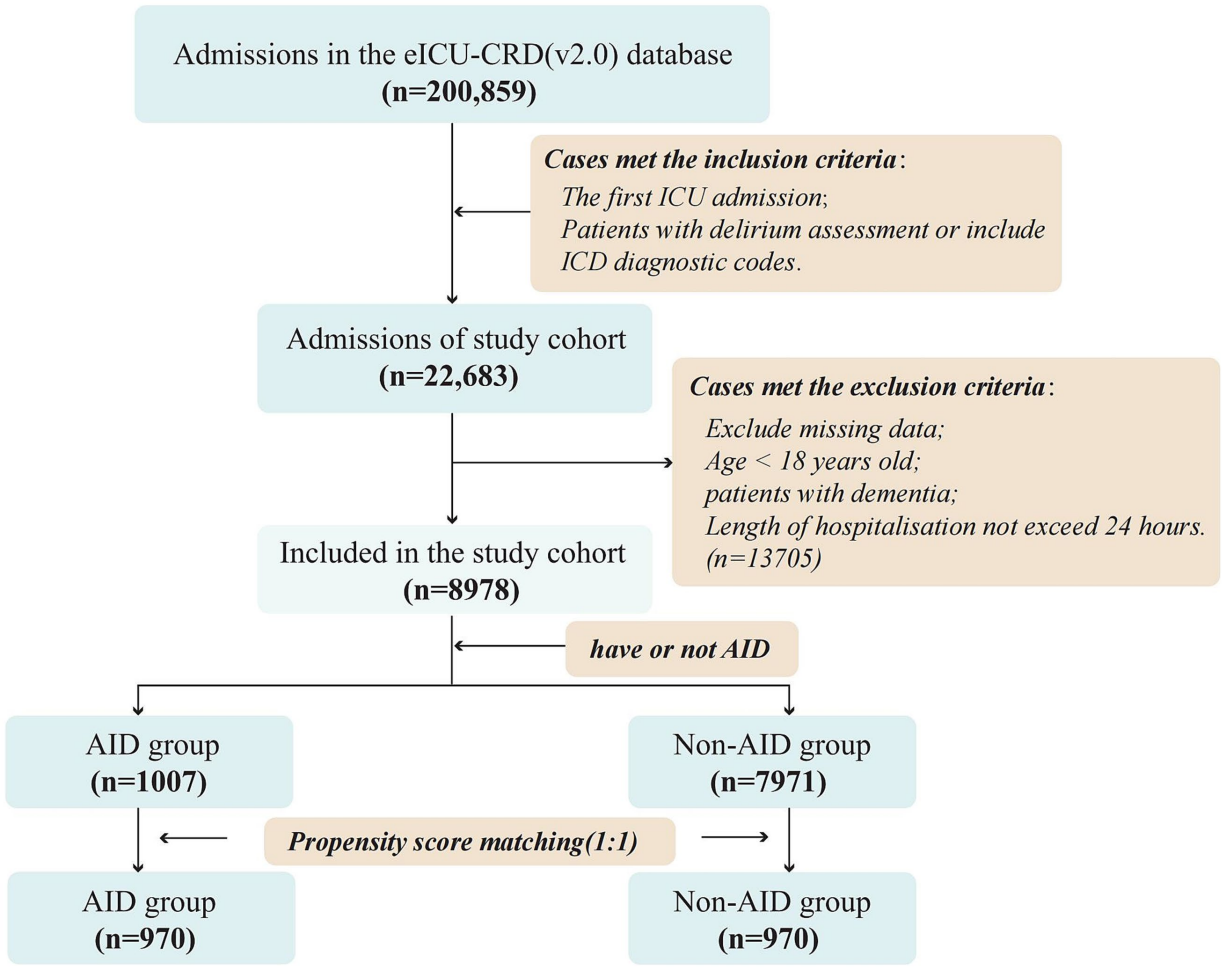
Flow chat and study design. eICU-CRD, eICU Collaborative Research Database; AID, autoimmune diseases; ICD code, International Classification of Diseases code; ICU, Intensive Care Unit.

We included patients admitted to the ICU for the first time who had at least one documented assessment of delirium (regardless of the result), and/or had a diagnosis of delirium based on ICD codes. Exclusion criteria were: (1) age <18 years; (2) total hospital stay <24 h; (3) a diagnosis of dementia to avoid misclassification with delirium; and (4) patients with missing values in selected variables to minimize bias. A total of 8,978 patients were included in the final analysis and were categorized into two groups based on the presence or absence of AID (Figure 1).

The primary outcome was the occurrence of delirium. Secondary outcomes included ICU mortality and ICU length of stay.

### 2.3 Definition of delirium and AID

Delirium in this study was defined by combining assessments using relevant tools with ICD codes. This multi-source approach aimed to improve diagnostic sensitivity and mitigate inter-center variability in assessment frequency. We searched the “diagnosis” table for ICD codes or diagnoses related to “delirium.” In addition, we extracted relevant bedside assessment data from the relevant nursing record tables, where tools such as the Confusion Assessment Method for the ICU (CAM-ICU) and the Intensive Care Delirium Screening Checklist (ICDSC) are routinely documented across participating ICUs. Positive findings from either source were used to define a delirium episode. The CAM-ICU and the ICDSC are the two tools recommended for diagnosing delirium in the ICU by the PADIS guidelines (1). A meta-analysis reported pooled sensitivities of 0.84 and 0.83 and specificities of 0.95 and 0.87 for CAM-ICU and ICDSC, respectively (21). Both tools are available in the eICU-CRD.

AID were identified based on ICD codes and included type 1 diabetes mellitus, multiple sclerosis, systemic sclerosis, systemic lupus erythematosus, amyloidosis, Crohn’s disease, ulcerative colitis, rheumatoid arthritis, dermatomyositis, psoriasis, and vasculitis. Relevant published studies were also referenced (22, 23). The proportions of each AID type among the AID cohort are shown in Supplementary Figure 3.

### 2.4 Variable extraction

Data were extracted using Navicat Premium (version 16.0) and SQL queries from the eICU GitHub repository.^1^ The following variables were included: demographics, vital signs, laboratory values, comorbidities, clinical interventions and medications, clinical scores, and indicators of disease severity.

The dependent variable was the occurrence of delirium. Additionally, a total of 31 independent variables were analyzed: Age, Gender, Race, Heart Rate, Mean Non-invasive Blood Pressure, Saturation of Peripheral Oxygen, White Blood Cell Count, Hemoglobin, Blood Urea Nitrogen, Anion Gap, Bicarbonate, Stroke, Hepatic Failure, Heart Failure, Chronic Kidney Disease, Sepsis-3, Hypertension, Chronic Obstructive Pulmonary Disease, Dialysis Treatment, Mechanical Ventilation, Steroid Use, Vasoactive Drug Use, Sedative Drug Use, Calcineurin Inhibitor Use, SOFA Score, GCS Score, APACHE IV, Bacterial Infection, Immunosuppression, Postoperative Status, and Bone Marrow Suppression.

For variables with multiple records during ICU stay, the value reflecting the most severe clinical condition was used. When multiple values were available for the same variable, the average was used for analysis. Full details are provided in Table 1.

**Table 1:** Baseline characteristics of autoimmune diseases (AID) patient cohorts with absolute standardized mean differences pre- and post-matching.

Characteristic	Before PSM					After PSM				
	All patients (*n* = 8,978)	Non-AID group (*n* = 7,971)	AID group (*n* = 1,007)	*P* value	SMD	All patients (*n* = 1,940)	Non-AID group (*n* = 970)	AID group (*n* = 970)	*P* value	SMD
Basic characteristics										
Age (year), median [IQR]	49.00 [38.00, 59.00]	49.00 [39.00, 59.00]	46.00 [34.00, 57.00]	<0.001	0.21	63.00 [53.00, 74.00]	64.00 [54.00, 73.00]	63.00 [52.00, 74.00]	0.62	0.028
Gender, No (%)										
Female	3,940 (43.9)	3,433 (43.1)	507 (50.3)	<0.001	0.146	969 (49.9)	485 (50.0)	484 (49.9)	1	0.002
Male	5,038 (56.1)	4,538 (56.9)	500 (49.7)			971 (50.1)	485 (50.0)	486 (50.1)		
Ethnicity, No (%)										
African American	782 (8.7)	688 (8.6)	94 (9.3)	0.036	0.129	195 (10.1)	105 (10.8)	90 (9.3)	0.004	0.19
Asian	105 (1.2)	93 (1.2)	12 (1.2)			24 (1.2)	12 (1.2)	12 (1.2)		
Caucasian	7,110 (79.2)	6,297 (79.0)	813 (80.7)			1,504 (77.5)	721 (74.3)	783 (80.7)		
Hispanic	445 (5.0)	394 (4.9)	51 (5.1)			112 (5.8)	62 (6.4)	50 (5.2)		
Native American	99 (1.1)	95 (1.2)	4 (0.4)			13 (0.7)	10 (1.0)	3 (0.3)		
Other/unknown	437 (4.9)	404 (5.1)	33 (3.3)			92 (4.7)	60 (6.2)	32 (3.3)		
Respiratory rate (bpm), median [IQR]	86.19 [75.92, 97.73]	85.80 [75.52, 97.22]	89.63 [79.51, 101.85]	<0.001	0.238	88.18 [78.10, 100.26]	87.04 [76.84, 98.40]	89.54 [79.26, 101.88]	<0.001	0.159
SPO2 (%), median [IQR]	97.23 [95.73, 98.54]	97.25 [95.75, 98.56]	97.08 [95.38, 98.40]	0.818	0.082	97.06 [95.52, 98.41]	97.00 [95.62, 98.41]	97.08 [95.38, 98.41]	0.845	0.012
NIBP (mmHg), median [IQR]	77.07 [69.86, 86.33]	77.04 [69.88, 86.22]	77.28 [69.64, 87.16]	0.498	0.049	77.37 [70.18, 87.24]	77.54 [70.40, 87.37]	77.27 [69.70, 87.16]	0.404	0.014
Laboratory										
WBC (K/mcL), median [IQR]	9.00 [6.50, 12.40]	8.90 [6.50, 12.30]	9.30 [6.60, 13.20]	0.006	0.067	9.20 [6.55, 12.81]	9.20 [6.60, 12.57]	9.30 [6.51, 13.10]	0.581	0.028
Platelet_min (median [IQR])	167.00 [119.00, 226.00]	166.00 [118.00, 224.00]	177.00 [125.00, 244.00]	<0.001	0.116	178.00 [127.00, 239.00]	180.00 [130.00, 235.75]	176.00 [125.00, 244.00]	0.667	0.025
Hematocrit (%), median [IQR]	30.50 [25.10, 35.90]	30.50 [25.10, 35.90]	30.80 [25.70, 36.05]	0.173	0.045	30.70 [25.40, 35.90]	30.50 [25.00, 35.70]	30.80 [25.80, 36.10]	0.239	0.054
Hemoglobin (g/dL), median [IQR]	10.10 [8.40, 11.80]	10.10 [8.40, 11.80]	10.10 [8.50, 11.90]	0.473	0.028	10.10 [8.40, 11.90]	10.00 [8.30, 11.80]	10.20 [8.50, 11.90]	0.35	0.043
Bun (mg/dL), median [IQR]	24.00 [16.00, 40.00]	24.00 [16.00, 39.00]	26.00 [16.00, 44.00]	0.015	0.091	26.00 [17.00, 44.00]	27.00 [17.00, 44.00]	26.00 [16.00, 44.00]	0.231	0.048
Bicarbonate (mmol/L), median [IQR]	24.00 [21.25, 26.75]	24.00 [21.46, 26.78]	23.50 [20.00, 26.50]	<0.001	0.181	23.75 [20.33, 26.50]	24.00 [20.50, 26.42]	23.55 [20.00, 26.50]	0.211	0.033
Aniongap, median [IQR]	10.50 [8.00, 14.00]	10.50 [7.90, 13.75]	12.25 [8.00, 14.88]	<0.001	0.145	11.20 [8.00, 14.88]	11.00 [8.00, 14.11]	12.13 [8.00, 14.88]	0.059	0.058
PTT(s), median [IQR]	32.00 [28.00, 41.00]	32.00 [28.00, 41.00]	32.20 [27.60, 41.00]	0.807	0.005	32.00 [27.60, 40.70]	32.00 [27.70, 40.70]	32.10 [27.60, 40.65]	0.776	0.04
INR, median [IQR]	1.20 [1.10, 1.50]	1.20 [1.10, 1.50]	1.20 [1.10, 1.50]	0.166	0.04	1.20 [1.10, 1.50]	1.20 [1.10, 1.44]	1.20 [1.10, 1.50]	0.324	0.016
PT(s), median [IQR]	14.50 [12.50, 17.60]	14.50 [12.50, 17.70]	14.30 [12.30, 17.10]	0.015	0.012	14.30 [12.20, 17.10]	14.20 [12.20, 17.20]	14.30 [12.30, 17.00]	0.966	0.005
Albumin (g/dL), median [IQR]	2.90 [2.75, 3.40]	2.90 [2.75, 3.45]	2.81 [2.70, 3.34]	0.001	0.095	2.81 [2.70, 3.40]	2.90 [2.70, 3.40]	2.81 [2.73, 3.35]	0.087	0.058
Comorbidities										
Heart Failure, No (%)	1,071 (11.9)	900 (11.3)	171 (17.0)	<0.001	0.164	326 (16.8)	167 (17.2)	159 (16.4)	0.671	0.022
Myocardial infarct, No (%)	502 (5.6)	451 (5.7)	51 (5.1)	0.484	0.026	101 (5.2)	54 (5.6)	47 (4.8)	0.87	0.015
Chronic kidney disease, No (%)	585 (6.5)	501 (6.3)	84 (8.3)	0.015	0.079	171 (8.8)	89 (9.2)	82 (8.5)	0.631	0.025
COPD, No (%)	907 (10.1)	733 (9.2)	174 (17.3)	<0.001	0.24	340 (17.5)	178 (18.4)	162 (16.7)	0.37	0.043
Diabetes, No (%)	1,207 (13.4)	858 (10.8)	349 (34.7)	<0.001	0.595	635 (32.7)	320 (33.0)	315 (32.5)	0.847	0.011
Hypertension, No (%)	1,429 (15.9)	1,100 (13.8)	329 (32.7)	<0.001	0.458	626 (32.3)	324 (33.4)	302 (31.1)	0.308	0.049
Stroke, No (%)	417 (4.6)	370 (4.6)	47 (4.7)	1	0.001	100 (5.2)	55 (5.7)	45 (4.6)	0.355	0.047
Hepaticfailure, No (%)	142 (1.6)	135 (1.7)	7 (0.7)	0.024	0.092	15 (0.8)	8 (0.8)	7 (0.7)	1	0.012
Metastaticcancer, No (%)	186 (2.1)	166 (2.1)	20 (2.0)	0.932	0.007	38 (2.0)	18 (1.9)	20 (2.1)	0.87	0.015
Sepsis-3, No (%)	1,275 (14.2)	963 (12.1)	312 (31.0)	<0.001	0.473	566 (29.2)	281 (29.0)	285 (29.4)	0.881	0.009
Interventions use/related evaluations										
Glucocorticoid (medication use), No (%)	2,264 (25.2)	2,037 (25.6)	227 (22.5)	0.042	0.071	450 (23.2)	230 (23.7)	220 (22.7)	0.628	0.024
Calcineurin inhibitor (medication use), No (%)	99 (1.1)	89 (1.1)	10 (1.0)	0.847	0.012	33 (1.7)	23 (2.4)	10 (1.0)	0.035	0.104
Vasoactive drug (medication use), No (%)	2,342 (26.1)	2,182 (27.4)	160 (15.9)	<0.001	0.282	325 (16.8)	165 (17.0)	160 (16.5)	0.808	0.014
Received dialysis, No (%)	513 (5.7)	442 (5.5)	71 (7.1)	0.062	0.062	136 (7.0)	69 (7.1)	67 (6.9)	0.929	0.008
Mechanical ventilation, No (%)	5,245 (58.4)	4,803 (60.3)	442 (43.9)	<0.001	0.332	864 (44.5)	433 (44.6)	431 (44.4)	0.964	0.004
Clinical scores/indicators of disease severity										
Sofa score, median [IQR]	15.00 [14.00, 16.00]	15.00 [14.00, 16.00]	15.00 [14.00, 16.00]	<0.001	0.14	15.00 [14.00, 16.00]	15.00 [14.00, 17.00]	15.00 [14.00, 16.00]	0.493	0.007
GCS score, median [IQR]	14.27 [12.50, 15.00]	14.33 [12.50, 15.00]	14.00 [12.49, 15.00]	0.001	0.055	14.04 [12.31, 15.00]	14.29 [12.00, 15.00]	14.00 [12.50, 15.00]	0.135	0.019
Apsiii score, median [IQR]	61.00 [47.00, 78.93]	61.00 [46.80, 78.00]	64.00 [47.40, 80.24]	0.023	0.064	62.00 [46.80, 79.00]	61.00 [46.00, 77.00]	63.50 [47.00, 80.00]	0.107	0.063
Postoperative status, No (%)	2,237 (24.9)	1,942 (24.4)	295 (29.3)	0.001	0.111	562 (29.0)	278 (28.7)	284 (29.3)	0.802	0.014
Bone marrow suppression, No (%)	402 (4.5)	291 (3.7)	111 (11.0)	<0.001	0.286	159 (8.2)	52 (5.4)	107 (11.0)	<0.001	0.208
Bacterial infection, No (%)	2,686 (29.9)	2,210 (27.7)	476 (47.3)	<0.001	0.412	802 (41.3)	351 (36.2)	451 (46.5)	<0.001	0.211
Immunosuppression, No (%)	300 (3.3)	264 (3.3)	36 (3.6)	0.73	0.014	66 (3.4)	32 (3.3)	34 (3.5)	0.9	0.011
Outcomes										
Death in ICU, No (%)	599 (6.7)	514 (6.4)	85 (8.4)	0.02	0.076	134 (6.9)	55 (5.7)	79 (8.1)	0.039	0.098
ICU LOS (days), median [IQR]	2.70 [1.54, 5.02]	2.57 [1.49, 4.79]	3.92 [1.92, 8.02]	<0.001	0.355	3.04 [1.77, 5.99]	2.66 [1.66, 4.75]	3.89 [1.91, 7.94]	<0.001	0.342
Delirium, No (%)	2,669 (29.7)	2,028 (25.4)	641 (63.7)	<0.001	0.833	848 (43.7)	225 (23.2)	623 (64.2)	<0.001	0.909

SPO2, Saturation of Peripheral Oxygen; NIBP, Non-invasive Blood Pressure; WBC, White Blood Cell; BUN, Blood urea nitrogen; INR, International Normalized Ratio; PT, Prothrombin Time; PTT, Partial Thromboplastin Time; COPD, Chronic Obstructive Pulmonary Disease; Sepsis-3, Third International Consensus Definition of Sepsis and Septic Shock; SOFA, Sequential Organ Failure Assessment; Apsiii, Acute Physiology and Chronic Health Evaluation IV; ICU LOS, ICU length of stay.

### 2.5 Statistical analysis

The sample size was based on the available data in the database. As a retrospective cohort study, it mainly relies on the analysis and summary of existing data, without prior statistical analysis schemes or statistical efficacy calculations.

Categorical variables were presented as counts (percentages) and compared using the chi-square test or Fisher’s exact test. Continuous variables were expressed as mean ± standard deviation or median [interquartile range (IQR)], depending on distribution and homogeneity of variance, and were compared using one-way ANOVA or Kruskal–Wallis tests, as appropriate.

PSM was employed to minimize baseline differences between groups. Matching was performed using a 1:1 nearest-neighbor algorithm without replacement, with a caliper of 0.02. Additionally, inverse probability of treatment weighting (IPTW) based on propensity scores was used to assign a weight to each patient (24). The standardized mean difference (SMD) was used to assess covariate balance before and after matching (25).

Cox proportional hazards regression models were used to estimate hazard ratios (HRs) and 95% confidence intervals (CIs) for the primary outcome. Univariate Cox regression analysis identified positive variables, followed by Least Absolute Shrinkage and Selection Operator (LASSO) regression analysis to select the potential predictors among the significant variables from univariate analysis (Supplementary Figures 1a,b, 2), followed by a stepwise method screening of these variables, and combined with clinically relevant routine variables for multivariate Cox multiple regression analysis.

Variance inflation factor (VIF) was used to detect multicollinearity, and variables with VIF > 5 were excluded (Supplementary Figure 2).

Competing risk analysis using the Fine and Gray method was applied to compare the cumulative incidence of delirium between AID and non-AID groups before and after PSM, considering death as a competing event (26, 27). For secondary outcomes, Kaplan–Meier analysis and log-rank tests were used to compare survival, and multivariable logistic regression was used for continuous outcomes with the same set of covariates. Mann–Whitney U tests and Hodges–Lehmann estimators were used to calculate median differences (MDs) and 95% CIs for continuous secondary outcomes.

Subgroup analyses in the matched cohort were stratified by categorical variables, including sex, interventions and medications (dialysis, mechanical ventilation, vasopressors, corticosteroids), comorbidities (chronic kidney disease, stroke, sepsis), and clinical states (immunosuppression, postoperative status, bone marrow suppression, bacterial infection). Interaction analyses were performed to evaluate effect modification between AID and continuous covariates across subgroups. STEPP (Subpopulation Treatment Effect Pattern Plot) analysis was used to explore treatment effect patterns and identify potential thresholds of differentiation (28, 29).

An *E*-value was calculated to assess the potential effect of unmeasured confounders, estimating the minimum strength of association that an unmeasured confounder would need to explain away the observed association between AID and delirium risk (30).

All statistical analyses were conducted using RStudio (v4.4.1), IBM SPSS Statistics (v25), and Stata (v16.0). Adobe Illustrator (2022) was used for figure generation. A two-sided *p*-value <0.05 was considered statistically significant.

## 3 Results

### 3.1 Baseline characteristics of the study cohort

A total of 200,859 patient records from the eICU-CRD (v2.0) were initially screened, from which 22,683 admissions met the inclusion criteria. After applying the exclusion criteria, 8,978 patients were included in the final study cohort. These were categorized into the AID group (*n* = 1,007) and the non-AID group (*n* = 7,971) based on the presence or absence of AID. The overall incidence of ICU delirium in the cohort was 29.7% (*n* = 2,669), and the ICU mortality rate was 6.7% (n = 599). Baseline characteristics before and after matching are detailed in Table 1.

Among the 1,007 ICU patients with AID, the most common diagnoses were Type 1 Diabetes Mellitus (about 31%), Multiple Sclerosis (about 18%), and Rheumatoid Arthritis (about 14%). Other identified conditions included Amyloidosis, Systemic Lupus Erythematosus, Crohn’s Disease, Ulcerative Colitis, Systemic Sclerosis, Vasculitis, Psoriasis and Dermatomyositis. A detailed breakdown of AID types and proportions is presented in Supplementary Figure 3.

**Figure 2:**
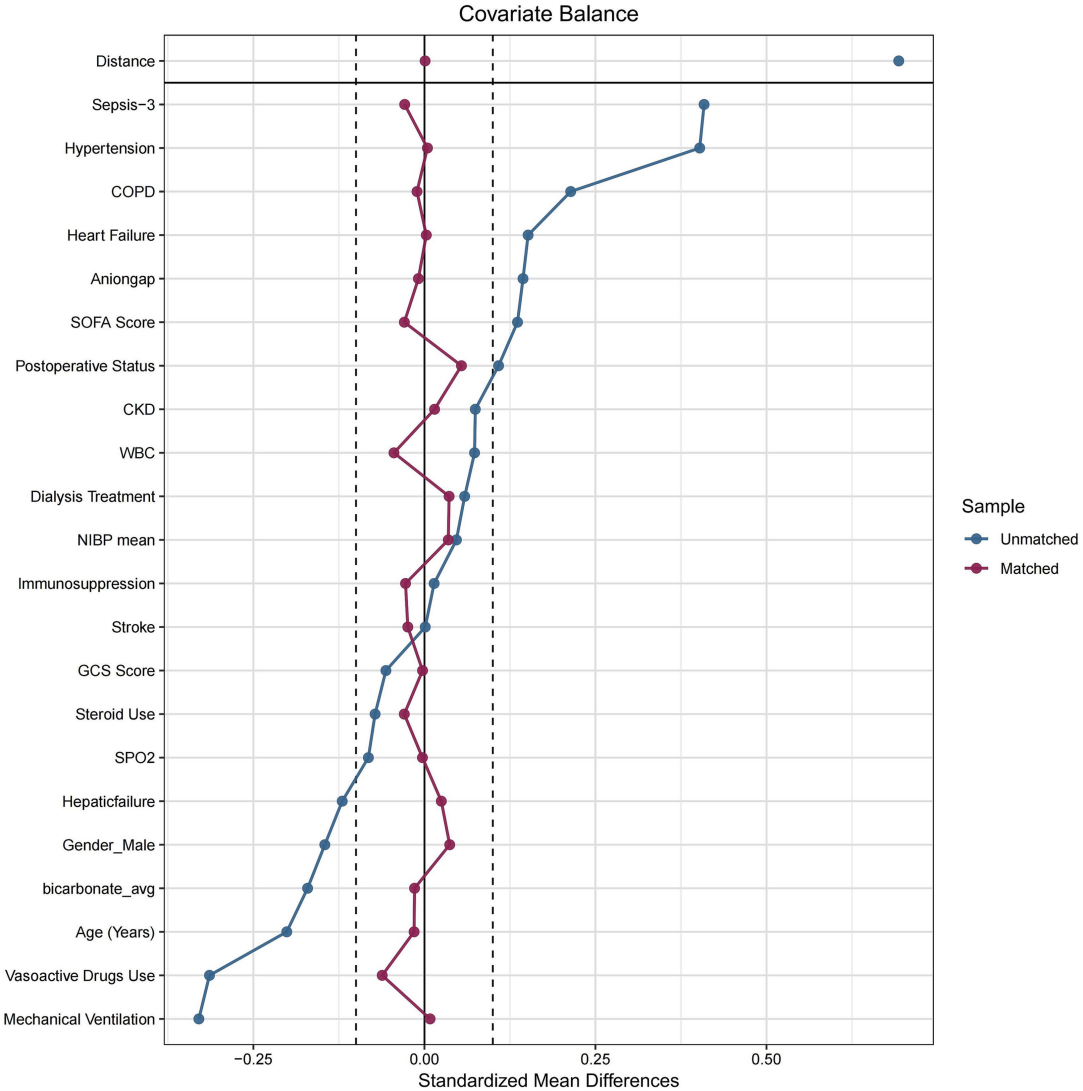
Baseline feature differences with pre-propensity and post-propensity score matching between two groups. Sepsis-3, third international consensus definition of sepsis and septic shock; COPD, Chronic Obstructive Pulmonary Disease; SOFA, sequential organ failure assessment; CKD, chronic kidney disease; WBC, White Blood Cell; NIBP, Non-invasive Blood Pressure; SPO2, Saturation of Peripheral Oxygen.

After PSM, 970 patients were included in each group, with good balance in baseline characteristics. The absolute value of all standardized mean differences (SMDs) were <0.1, indicating adequate matching. The distribution of SMDs before and after matching is visualized in Figure 2.

### 3.2 Association between AID and delirium

Before PSM, univariate Cox regression showed a significant positive association between AID and the risk of delirium (HR: 1.879; 95% CI: 1.718–2.055; *p* < 0.001). This association remained significant after adjusting for covariates in the multivariate model (HR: 2.171; 95% CI: 1.957–2.406; *p* < 0.001).

After PSM, AID was still significantly associated with an increased risk of delirium in both univariate (PSM-adjusted, HR: 1.746; 95% CI: 1.496–2.037 *p* < 0.001; IPTW-adjusted, HR: 1.588; 95% CI: 1.354–1.862; *p* < 0.001) and multivariate analyses (PSM-adjusted HR: 2.376; 95% CI: 2.025–2.788; *p* < 0.001). Similar results were obtained using the IPTW method (HR: 2.365; 95% CI: 2.018–2.773; *p* < 0.001). The results are detailed in Table 2.

**Table 2:** The relationship between autoimmune diseases (AID) and delirium incidence before and after propensity score matching.

	HR, 95%CI	*P* value
Univariate model		
Crude model	1.879 (1.718–2.055)	<0.001
PSM	1.746 (1.496–2.037)	<0.001
IPTW	1.588 (1.354–1.862)	<0.001
Multivariate model		
Crude model	2.171 (1.957–2.406)	<0.001
PSM	2.376 (2.025–2.788)	<0.001
IPTW	2.365 (2.018–2.773)	<0.001

PSM, Propensity Score Matching; IPTW, Inverse Probability of Treatment Weighting; HR, hazard ratios; 95%CI, 95% confidence intervals.

### 3.3 Secondary outcomes

Kaplan–Meier curves revealed no significant difference in ICU mortality between the two groups (log-rank test, *p* > 0.05; shown in Supplementary Figure 4).

The median length of ICU stay was 3.89 days (IQR, 1.90–7.94) in the AID group and 2.65 days (IQR, 1.65–4.75) in the non-AID group. The median length of ICU stay was longer in the AID group than in the non-AID group. AID was associated with prolonged length of ICU stay (MD: 6.5; 95% CI: 3.7–11.3; *p* < 0.001). The results are detailed in Table 3.

**Table 3:** The association of autoimmune diseases (AID) and continuity secondary outcomes in the PSM matched cohort.

Secondary outcome	Exposures	Non-AID	AID	MD (95%CI)	*P*-value
		*n* = 970	*n* = 970		
ICU Los (days), median (IQR)	AID	2.65 (1.65–4.75)	3.89 (1.90–7.94)	6.5 (3.7–11.3)	<0.001

IQR, inter quartile range; AID, Autoimmune Diseases; Los, Length of stays; 95%CI, 95% confidence intervals; MD with 95%CI was calculated using Hodges-Lehmann extimator.

### 3.4 Competing risk analysis

**Figure 3:**
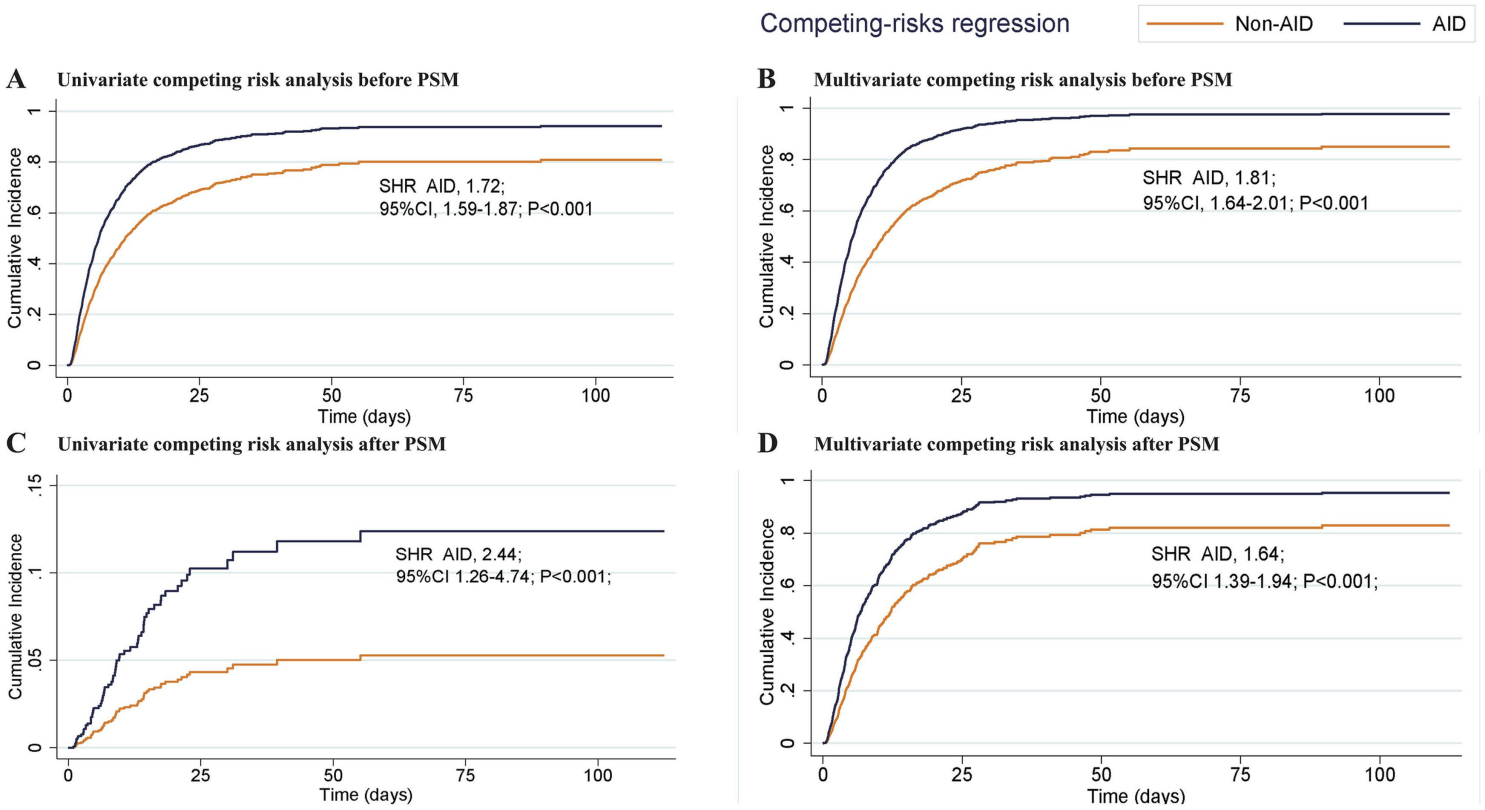
Cumulative incidence curves of delirium within the ICU for patients with and without autoimmune diseases. **(A, B)** shows the curves before PSM; **(C, D)** shows the curves after PSM AID, autoimmune diseases; ICU, Intensive Care Unit; PSM, propensity score matching; SHR, Subhazard Ratio; 95% CI, 95% confidence intervals.

A Fine and Gray competing risk model was applied to account for ICU death as a competing event for delirium. After adjusting for this competing risk, AID remained significantly associated with a higher cumulative incidence of delirium (*p* < 0.001, see Table 4 for details). Corresponding cumulative incidence curves are shown in Figure 3. Univariate and multivariate competing risk regression models before and after PSM confirmed the robustness of the findings (Figures 3A,B shows the curves before PSM; Figures 3C,D shows the curves after PSM).

**Table 4:** Univariate and multivariate competing risk analysis before and after propensity score matching.

	SHR, 95%CI	*P* value
Before PSM		
Univariate competing risk analysis	1.72 (1.59–1.87)	< 0.001
Multivariable competing risk analysis	1.81 (1.64–2.01)	< 0.001
After PSM		
Univariate competing risk analysis	2.44 (1.26–4.74)	< 0.001
Multivariable competing risk analysis	1.64 (1.39–1.94)	< 0.001

PSM, Propensity Score Matching; IPTW, Inverse Probability of Treatment Weighting; SHR, Subhazard Ratio; 95%CI, 95% confidence intervals.

### 3.5 Subgroup analyses

**Figure 4:**
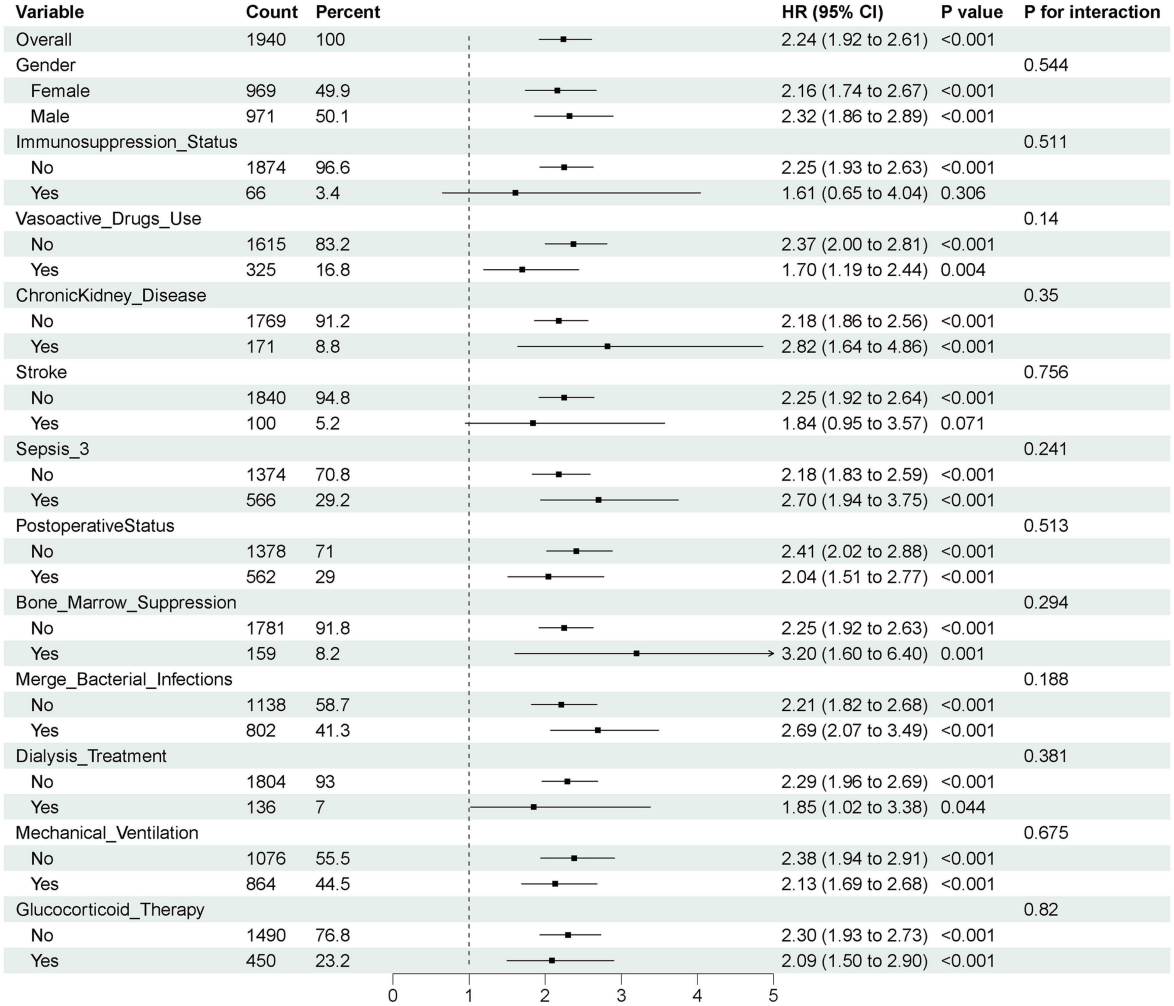
Subgroup analyses for outcome in the propensity score matching cohort. Sepsis-3, Third International Consensus Definition of Sepsis and Septic Shock; HR, hazard ratio; 95% CI, 95% confidence intervals.

Subgroup analyses and interaction tests revealed no significant heterogeneity in the association between AID and delirium across different patient subgroups (all *p*-value for interaction > 0.05), as shown in Figure 4.

### 3.6 Sensitivity analyses

*E*-value analysis showed that an unmeasured confounder would need to have a risk ratio of at least 2.95 (upper confidence limit: 2.71) to fully explain the observed association between AID and delirium (Table 5). This suggests that the influence of potential unmeasured confounding is unlikely to negate the observed findings.

**Table 5:** *E*-value for association between autoimmune diseases (AID) and delirium.

Exposure	Outcomes	*E*-value	Upper limit of 95% CI
Autoimmune diseases	Delirium	2.95	2.71

## 4 Discussion

This study, based on the multicenter database (eICU-CRD), systematically evaluated the impact of AID on the risk of delirium in critically ill patients using PSM and IPTW method. The results consistently demonstrated that AID was significantly associated with an increased risk of delirium, serving as an independent risk factor in both the original and matched cohorts through Cox regression analysis, as well as in competing risk models accounting for ICU mortality. In contrast to previous studies that focused on traditional risk factors such as infections, aging, sedation, or postoperative status (31), our findings suggest that AID may represent an under-recognized but clinically important contributor to ICU delirium risk. This is in line with recent research indicating systemic immune dysregulation may as a central driver of neuropsychiatric manifestations in acute care settings (14, 15). Subgroup analysis showed that there was no significant interaction observed between AID and delirium risk in subgroups of different genders, underlying diseases, treatment measures, or immune status, indicating a certain degree of consistency in its impact across different populations.

Although prior research on the association between AID and CNS complications has largely been limited to isolated case reports—for example, an early case describing a patient with systemic lupus erythematosus presenting with subacute delirium, suggesting the possibility of immune-mediated neuropsychiatric manifestations—large-scale empirical data have been lacking (32). In this context, our study fills a critical knowledge gap by providing the first evidence from a large, multicenter cohort using robust PSM-based methodology to assess the AID-delirium relationship. Similarly, recent PSM-based studies on alcohol use disorder (AUD) have shown AUD to be an independent risk factor for delirium, further underscoring the significance of chronic systemic disturbances in delirium pathophysiology (33).

It is well established that delirium is tightly linked to CNS inflammation (18). AID may contribute to this process through multiple mechanisms, including chronic systemic inflammation, microglial activation, neurotransmitter imbalances, and autoantibody-mediated neurotoxicity. Patients with AID often exhibit chronic systemic inflammation, and the intermittent increase in inflammation may potentially promote disease progression and systemic damage (34). At the genetic level, studies have shown considerable overlap in susceptibility loci between AID and chronic inflammatory states, supporting a shared pathophysiological basis (35). Moreover, systemic inflammation can alter the function of the blood–brain barrier (BBB), which plays a key role in maintaining the specialized CNS environment and mediating CNS-periphery communication (36). Such alterations may be either disruptive or non-disruptive, but they nevertheless impact CNS homeostasis (36). Clinically observable syndromes such as sickness behavior and delirium are associated with CNS dysfunction (18, 36).

Infections—particularly severe infections like sepsis—are known precipitants of delirium, primarily through CNS inflammation triggered by systemic immune responses (37). In the context of AID such as multiple sclerosis, chronic systemic inflammation can activate microglia (38–40), which may excessively prune synapses and release neurotoxic substances, ultimately leading to neuronal dysfunction and synaptic disorganization—mechanisms that may predispose to delirium. Additionally, common cytokine imbalances in AID patients, especially the elevation of pro-inflammatory factors such as IL-1β, IL-6, TNF-α, have been found to be closely related to the occurrence of delirium (41, 42). Beyond cytokine-mediated effects, certain AID-related autoantibodies may cross the BBB and directly damage CNS tissue. For example, antibodies against neuronal surface or synaptic proteins, such as anti-NMDA receptor and anti-LGI1 antibodies, have been associated with behavioral changes, cognitive dysfunction, and delirium-like manifestations in autoimmune encephalitis (12, 16).

Furthermore, previous studies have shown that AID patients seem to be more prone to sleep disturbances (43, 44), and sleep disorders share many common symptoms with delirium (45). A systematic review of 12 studies conducted in 2018 found a strong correlation between sleep disorders and the occurrence of postoperative delirium, which is related to pre-existing sleep disorders (46). When abnormal immune reactions caused by autoimmune diseases attack the CNS region (neuronal structure and neurotransmitter system) that regulates sleep, corresponding sleep symptoms will appear, and this association is complex and multifactorial (44). Similarly, inflammation-induced neurotransmitter imbalances—particularly involving the acetylcholine-dopamine axis—play a pivotal role in the pathophysiology of delirium. This involves altered connectivity among neural networks and disrupted neurotransmitter release patterns (31).

Although AID was found to increase the risk of ICU delirium in our study, it was not significantly associated with ICU mortality. This suggests that clinical attention in AID patients should be paid to the neurological function status, rather than being limited to traditional outcome measures. Despite being a reversible condition, delirium has been consistently associated with long-term cognitive impairment, prolonged hospitalization, and increased healthcare resource utilization (5, 7). Early identification of high-risk individuals is therefore essential for timely intervention.

Based on our findings, we propose that AID status should be incorporated into delirium risk stratification in the ICU setting. In addition to conventional delirium screening tools such as CAM-ICU and ICDSC, incorporating a patient’s autoimmune background—alongside inflammatory biomarkers such as the systemic immune-inflammation index—may facilitate a multidimensional approach to risk prediction (47, 48). With the growing application of immune-targeted therapies, agents such as the IL-6 inhibitor (tocilizumab), which has shown potential benefit in managing delirium among critically ill COVID-19 patients (49), may warrant future investigation in AID populations for both prevention and treatment. Although the observed effect size was moderate, it may still carry clinical significance given the high baseline incidence and adverse outcomes of ICU delirium. Our findings suggest that autoimmune disease status may help identify a subset of patients at elevated neuropsychiatric risk, potentially supporting more targeted screening and preventive strategies. In depth studies are needed to determine clinically meaningful thresholds and metrics such as number needed to screen (NNS) or number needed to treat (NNT) to inform practical ICU implementation.

### 4.1 Strengths and limitations

This study has several notable strengths. By leveraging a large, multicenter database and employing multiple analytic approaches—including PSM, IPTW, and competing risk models—we minimized intergroup variability and common biases, thereby enhancing the robustness of our conclusions. Furthermore, we accounted for key confounders such as immunosuppression, myelosuppression, postoperative status, and calcineurin inhibitor use, ensuring the reliability of our results. Nevertheless, limitations remain. Despite extensive confounding control—including *E*-value analysis—residual confounding inherent to retrospective designs cannot be fully excluded. The eICU-CRD also lacks detailed data on AID subtypes and disease activity, limiting our ability to perform granular subgroup analyses. The observed heterogeneity in AID subtypes may contribute to the varying risk of ICU delirium among affected patients. Conditions like systemic lupus erythematosus and multiple sclerosis involve neuroinflammation, potentially increasing delirium susceptibility. Moreover, immunosuppressive treatment strategies differ markedly across AID types. We noticed that the eICU-CRD comes from ICUs in the United States, so the generalizability of the research results is limited by non Western or low resource environments, where AID prevalence, ICU practices, and delirium detection vary. Finally, delirium diagnoses were based on CAM-ICU, ICDSC assessments, and ICD codes, due to the multicenter nature of the eICU-CRD, the frequency and consistency of assessments of delirium may vary across institutions. Although we attempted to minimize misclassification by integrating both diagnostic codes and standardized nursing assessments, variability in documentation remains an inherent limitation of secondary database research. Future high-quality prospective studies and randomized controlled trials are needed to further delineate the specific relationships between AID subtypes, autoantibody profiles, and delirium, and to evaluate the potential of individualized immunomodulatory strategies in improving neuropsychiatric outcomes.

## 5 Conclusion

This study represents the first large-scale evidence demonstrating a significant association between AID and ICU delirium, emphasizing the clinical importance of recognizing immune disease status in delirium management. Through a systematic comparison of our findings with current research, this study not only confirms AID as an independent risk factor for ICU delirium, but also highlights the biological link between inflammation–immune dysregulation and neurological impairment. These insights provide a clear direction for future efforts in risk prediction, mechanistic exploration, and targeted intervention.

## Data Availability

Publicly available datasets were analyzed in this study. This data can be found here: The data that support the findings of this study are available from the eICU-CRD, but restrictions apply to the availability of these data, which were used under license for the current research and so are not publicly available. However, available from the authors (STH) upon reasonable request and with the permission of the holder of the database. The access policy and procedures of eICU-CRD dataset are available at https://physionet.org/content/eicu-crd/2.0/.

## Footnotes

^1^ https://github.com/MIT-LCP/eicu-code

## Glossary

ICU: Intensive care unit
AID: Autoimmune diseases
CNS: Central nervous system
eICU-CRD: eICU Collaborative Research Database
PSM: Propensity score matching
STROBE: Strengthening the reporting of observational studies in epidemiology
ICD: International classification of diseases
Apsiii: Acute physiology and chronic health evaluation IV
SOFA: Sequential organ failure assessment
Sepsis-3: Third international consensus definition of sepsis and septic shock
CAM-ICU: Confusion assessment method for the ICU
ICDSC: Intensive care delirium screening checklist
SMD: Standardized mean difference
IQR: Interquartile range
HR: Hazard ratio
95% CI: 95% confidence intervals
LASSO: Least absolute shrinkage and selection operator
VIF: Variance inflation factors
MD: Median difference
IPTW: Inverse probability of treatment weighting
